# Andrographolide stabilized-silver nanoparticles overcome ceftazidime-resistant *Burkholderia pseudomallei*: study of antimicrobial activity and mode of action

**DOI:** 10.1038/s41598-022-14550-x

**Published:** 2022-06-23

**Authors:** Saengrawee Thammawithan, Chanon Talodthaisong, Oranee Srichaiyapol, Rina Patramanon, James Andell Hutchison, Sirinan Kulchat

**Affiliations:** 1 Department of Biochemistry, Faculty of Science, Khon Kaen University, Khon Kaen, 40002 Thailand; 2 Protein and Proteomics Research Center for Commercial and Industrial Purposes, Khon Kaen University, Khon Kaen, 40002 Thailand; 3 Materials Chemistry Research Center, Department of Chemistry and Center of Excellence for Innovation in Chemistry, Faculty of Science, Khon Kaen University, Khon Kaen, 40002 Thailand; 4 School of Chemistry, The University of Melbourne, Parkville, VIC 3010 Australia

**Keywords:** Microbiology, Antimicrobial resistance

## Abstract

*Burkholderia pseudomallei* (*B. pseudomallei*) is a Gram-negative pathogen that causes melioidosis, a deadly but neglected tropical disease. *B. pseudomallei* is intrinsically resistant to a growing list of antibiotics, and alternative antimicrobial agents are being sought with urgency. In this study, we synthesize andrographolide-stabilized silver nanoparticles (andro-AgNPs, spherically shaped with 16 nm average diameter) that show excellent antimicrobial activity against *B. pseudomallei*, including ceftazidime-resistant strains, being 1–3 orders of magnitude more effective than ceftazidime and 1–2 orders of magnitude more effective than other green-synthesized AgNPs. The andro-AgNPs are meanwhile non-toxic to mammalian cell lines. The mode of action of Andro-AgNPs toward *B. pseudomallei* is unraveled by killing kinetics, membrane neutralization, silver ions (Ag^+^) release, reactive oxygen species (ROS) induction, membrane integrity, and cell morphology change studies. The antimicrobial activity and mode of action of andro-AgNPs against *B. pseudomallei* reported here may pave the way to alternative treatments for melioidosis.

## Introduction

Melioidosis is a clinically diverse disease caused by the facultative intracellular Gram-negative bacterium, *Burkholderia pseudomallei* (*B. pseudomallei*)^1^. The disease is a serious but neglected tropical disease of Southeast Asia, India, and Northern Australia^2^, and increasing reports of the condition are occurring worldwide. It has been estimated that there are 165,000 human melioidosis cases per year worldwide, accounting for 89,000 deaths^3^. *B. pseudomallei* has been called the “great mimicker” because its effects span a clinical spectrum ranging from pneumonia, localised cutaneous lesion, bacteremia without evident focus, septic arthritis and osteomyelitis, to severe sepsis with multiple organ abscesses^4^. This makes diagnosis of melioidosis difficult which may mask its prevalence^5^. Indeed, data for melioidosis are non-existent in many affected countries due to the lack of diagnostic microbiology facilities and a lack of familiarity of doctors and laboratory staff with its clinical and bacteriological features.

Melioidosis infection can occur in both humans and animals, usually resulting from exposure through broken skin, inhalation or ingestion of *B. pseudomallei*^6^. Exposure results in acute infections (85%) with high rates of mortality (10–50%) and relapse (5–28%)^7^. There is no approved human vaccine against *B. pseudomallei*. The current recommended melioidosis treatment requires β‑lactam antibiotics (such as ceftazidime, meropenem, imipenem and co‑amoxiclav). However, *B. pseudomallei* is intrinsically resistant to penicillin, ampicillin, first-generation and second-generation cephalosporins, gentamicin, tobramycin, and streptomycin^8^. Furthermore, resistant of *B. pseudomallei* toward ceftazidime and co-amoxiclav have emerged, leading to treatment failure^9,10^. *B. pseudomallei* remains susceptible to carbapenems (meropenem, imipenem) but excessive use of carbapenems can give rise to resistance, as has occurred with other antibiotics. Therefore, alternative antimicrobial agents are urgently required, and there is a need for a focus on promising new technologies, such as nanotechnology.

Silver nanoparticles (AgNPs) are metallic silver particles with a size distribution in the range 1–100 nm^11^. They show physical and chemical properties that differ substantially from bulk silver, importantly here, their biological activity depends on size, shape, and capping agent^12^. They have been applied for diverse applications important for human health including in medicinal devices, wound dressing, dentistry, water treatment, agriculture and medicine^13,14^. AgNPs display activity against a broad spectrum of bacteria, fungi, and viruses^15^. In particular, AgNPs can effectively inhibit drug-resistant bacteria^16^. Furthermore, this inhibition occurs through multiple modes of action such that development of resistance to AgNPs is very unlikely compared to conventional antibiotics^17^. Thus, AgNPs could be an alternative agent to fight antibiotic resistant bacteria, such as *B. pseudomallei*.

Aqueous wet chemical synthesis of AgNPs typical involves reduction of a silver salt to nucleate growing nanoparticles, followed by binding charged ligands to the particle surface, thus stabilizing them in the nano-size domain via electrostatic repulsion. Green syntheses employ natural products as reductants and surface binding agents, having the advantage of being cost-efficient, eco-friendly, and biocompatible, but also with the possibility of conferring their own biological activity to the nanoparticle via the stabilizing ligand^18^. Plants such as green and black tea, *Ocimum sanctum*, *Piper nigrum*, *Musa paradisiaca*, *Boerhavia diffusa* and *Mimusops elengi* have been used to synthesize AgNPs^19^. A medicinal plant of interest in this regard is *Andrographis paniculata* (*A. paniculata*), which is widely distributed in southern and southeast Asia and has been used by humans for centuries, for example in the Ayurvedic tradition. It contains a major bioactive chemical constituent known as andrographolide. Andrographolide is a diterpene lactone that shows anti-inflammatory, antioxidant, anticancer, and antimicrobial activities^20^. In previous studies, AgNPs synthesized using *A. paniculata* extract were reported to have antimicrobial activity against many types of bacteria and fungi including *Escherichia coli*, *Staphylococcus aureus*, *Salmonella typhimurium*, *Pseudomonas aeruginosa*, *Klebsiella pneumonia*, *Enterococcus faecalis*, *Candida albicans*, *Botrytis cinerea*^21,22^.

Herein, we evaluate antimicrobial effect and mode of action of andrographolide stabilized-AgNPs (andro-AgNPs) against *B. pseudomallei*, and against *E. coli* as a Gram-negative reference strain. The andro-AgNPs are synthesized using *A. paniculata* extract, characterized, then assessed for antimicrobial activity against three isolations of *B. pseudomallei*, including ceftazidime-resistant strains, with excellent results (MIC and MBC of 0.5 µg/mL and 8 µg/mL for all strains, 1–3 orders of magnitude lower than for ceftazidime, and 1–2 orders of magnitude lower than for reports of other green-synthesized AgNPs). However, the andro-AgNPs are shown to be non-toxic to mammalian Vero and MCF-7 cell lines at 100 µg/mL. The mode of action of Andro-AgNPs toward *B. pseudomallei* is unraveled by killing kinetics, membrane neutralization, silver ions (Ag^+^) release, reactive oxygen species (ROS) induction, membrane integrity, and cell morphology change studies. The antimicrobial activity and mode of action of andro-AgNPs against *B. pseudomallei* reported here might pave the way to alternative treatments for melioidosis.

## Results and discussion

### Synthesis and characterization of andro-AgNPs

**Figure 1:**
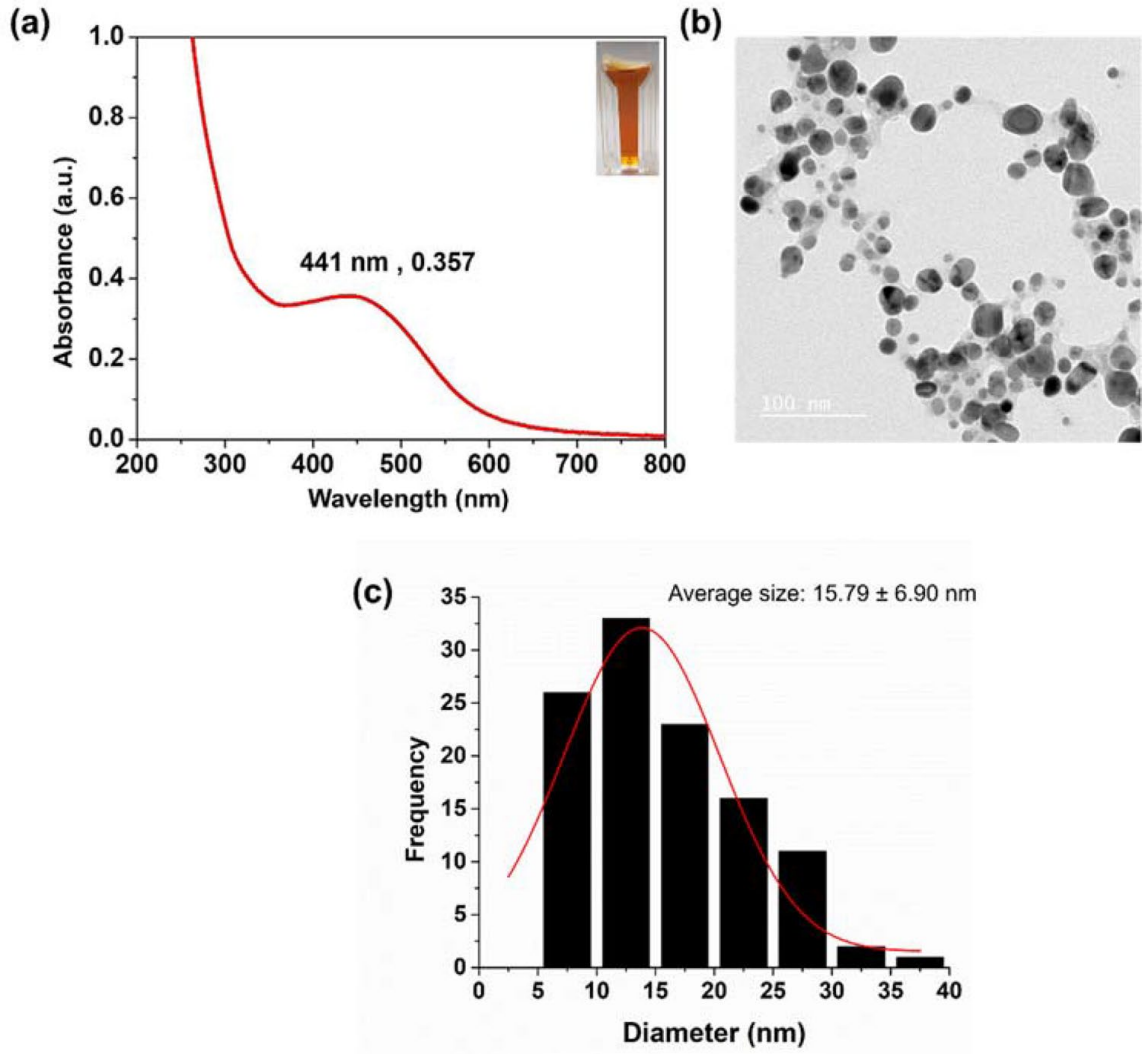
Physicochemical characterization of silver nanoparticles: (**a**) Extinction spectrum of an aqueous sol of andro-AgNPs, the band peaking at 441 nm is attributed to surface plasmon resonances giving a distinctive brown colour (see photograph of the solution inset); (**b**) Transmission Electron Microscopy (TEM) image of the andro-AgNPs; (**c**) histogram of the diameters of 112 andro-AgNPs from TEM studies, the average diameter was 15.79 ± 6.90 nm.

Andro-AgNPs were synthesized by reduction of silver nitrate in an aqueous andrographolide broth obtained from a commercial source of *A. paniculata* (see Materials and methods section). The absorption spectrum of the resultant andro-AgNPs is presented in Fig. 1a, the brown color of the solution is due to the characteristic absorption and scattering of localized surface plasmon resonances (SPR) peaking at 441 nm^23^. Fourier transform infrared (FTIR) spectroscopy shows that the functional groups present in purified andrographolide, and in *A. paniculata*-derived andrographolide capsules, are also present on the surface of the as-synthesized andro-AgNPs (see SI, Fig. S1). X-ray diffraction (XRD) studies suggest that the andro-AgNPs are highly crystalline, face centered cubic (fcc) metallic silver with average crystallite size of 3.82 nm calculated using the Scherrer equation (see SI, Fig. S2).

The size and morphology of the andro-AgNPs were also investigated by transmission electron microscopy (TEM), revealing distinct spherical but also irregularly-shaped nanoparticles (Fig. 1b). The distribution of particle sizes is relatively broad with a diameter range of 5–40 nm and an average size of 15.79 ± 6.90 nm (n = 112, Fig. 1c, analyzed using the Image J program). The hydrodynamic size of the andro-AgNPs was estimated from dynamic light scattering (DLS, Fig. S3a). The particle size distribution curve exhibits one peak, corresponding to an average particle diameter of 93.12 ± 3.76 nm with a polydispersity index (PDI) of 0.339 (the PdI estimates the particle size-uniformity in solution, with a value of 0.1 being considered monodisperse)^24^. This relatively large polydispersity correlates with the particle size/shape irregularity seen in the TEM images (Fig. 1b). The larger NP diameter determined by DLS may reflect solution aggregation of andro-AgNPs (see below) but also the stabilizing andrographolide layer/hydration sphere which is not measured by TEM. This result is similar to green synthesis of AgNPs reported by Chartarrayawadee et al.^25^. Zeta potential measurements were undertaken next to examine the stability of the silver suspensions (Fig. S3b)^26^. This technique measures particle mobility in an electric field and depends on the electrical potential at the junction of the diffuse ion layer surrounding the particle surface and the bulk solution^27^. As a rule, a suspension with zeta potential less than ± 30 mV can be unstable, resulting in particles settling out of solution^28^. The andro-AgNPs have a zeta potential of −17.26 ± 0.99 mV, nevertheless, we did not observe significant settling or precipitation over ten months.

### Cytotoxicity of AgNPs to mammalian Cells

**Figure 2:**
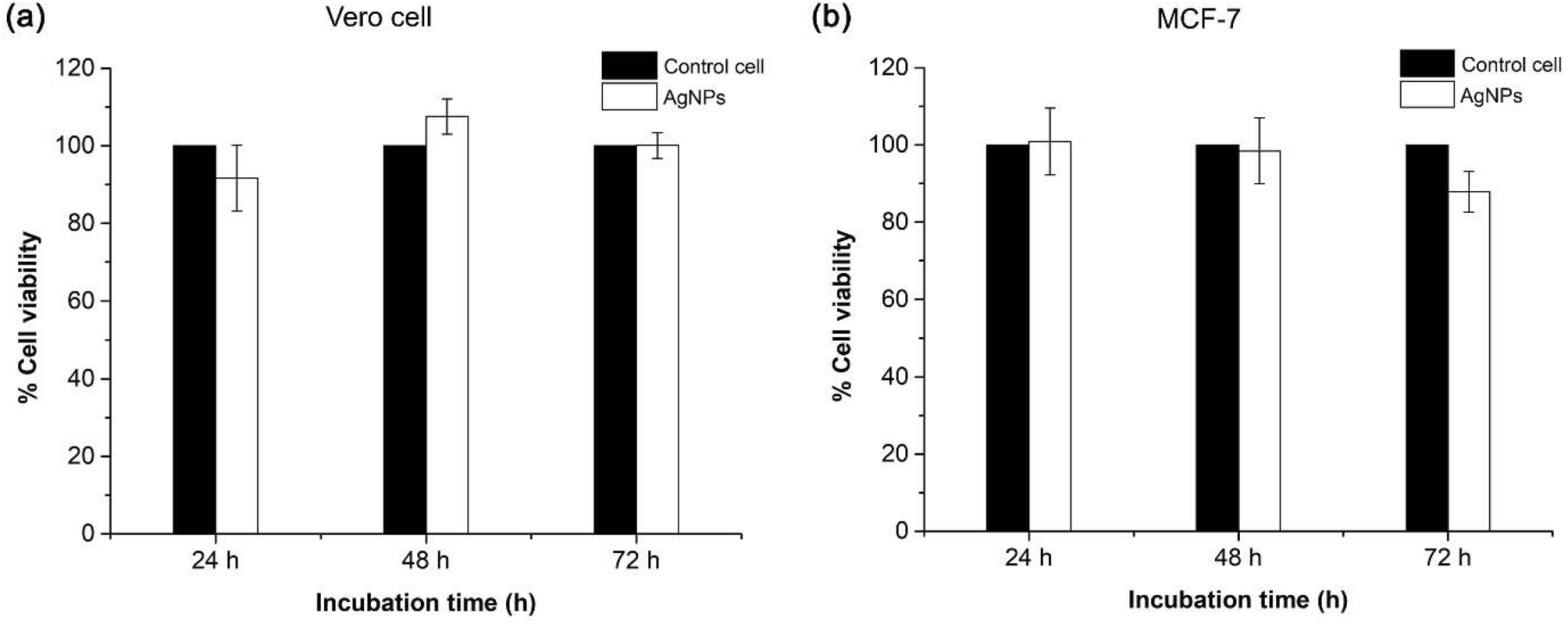
Cytotoxicity of andro-AgNPs towards normal (Vero cell) and cancerous (MCF-7) mammalian cell lines, evaluated by MTT assay. Vero cell and MFC-7 were incubated with andro-AgNPs at 100 µg/mL for 24, 48, and 72 h. The same cells without andro-AgNPs were monitored as a control for 100% viability. The data represent mean value ± SD (error bar) from two independent experiments carried out in triplicate (*n* = 6).

AgNPs feature well-known antimicrobial activity, however their effects on mammalian cells, which is relevant for clinical applications, are less known. Size, shape, and surface chemistry are key factors that mediate cellular response to AgNPs^29^. One feature of green syntheses of AgNPs using biological compounds such as tannin, curcumin, etc., is their potential to alleviate cytotoxicity^30,31^. Before antimicrobial assay, the cytotoxicity of the andro-AgNPs against Vero cell (monkey kidney epithelial cells) and MCF-7 breast cancer cell lines was evaluated in vitro via MTT assay (see "Materials and methods" section). Vero cell and MCF-7 lines were incubated with 100 µg/mL andro-AgNPs stock solution for 72 h (100 µg/mL is the maximum concentration of andro-AgNPs in the synthesized stock solution). At 24 h, 48 h, and 72 h of incubation, cell viability was determined and compared to untreated controls. The viability of Vero and MCF-7 cells treated with andro-AgNP was not significantly different to the untreated controls, even after 72 h incubation (Fig. 2).

These results can be compared to those of Selvam et al.^32^ who showed that AgNPs synthesized from *A. paniculata* extract showed activity against neuroblastoma cells, but were not cytotoxic to Vero cell lines with a CC_50_ (50% cytotoxicity concentration) value of 329.29 μg/ml. Our andro-AgNPs showed a lower cytotoxic effect than another previous report that found a maximum non-toxic dose value of 31.25 μg/mL in Vero cells^33^. In another report, AgNPs synthesized by *A. paniculata* extract showed cytotoxic effect against MDA-MB-453 breast cancer cells^34^. The non-toxicity of our andro-AgNPs towards both normal and cancerous mammalian cell lines might be caused by variations of the plant extract and synthesis process employed.

### Bacterial susceptibility to antimicrobial agents

The in vitro antimicrobial activity of andro-AgNPs against *B. pseudomallei* was tested next, and compared to ceftazidime (CAZ) antibiotic using a resazurin colorimetric assay and plate count method. The minimum inhibitory concentration (MIC) and minimum bactericidal concentration (MBC) of andro-AgNPs against three clinical isolates of *B. pseudomallei* (CAZ non-resistant and CAZ resistant isolates) and *E. coli* O157:H7 (a Gram-negative reference) are shown in Table 1. The MIC and MBC values of AgNPs against the three isolates of *B. pseudomallei* were equal at 0.5 µg/mL and 8 µg/mL, respectively. Whereas the MIC and MBC values of ceftazidime against *B. pseudomallei* were in the range of 32–256 µg/mL and 128–1024 µg/mL, respectively. The MIC and MBC values of andro-AgNPs against *E. coli* O157:H7 were 2 µg/mL and 8 µg/mL, respectively. The MIC and MBC values of ceftazidime against *E. coli* O157:H7 were 2 µg/mL and 4 µg/mL, respectively.

**Table 1:** The minimum inhibitory concentration (MIC) and minimum bactericidal concentration (MBC) of antimicrobial agents (andro-AgNPs and ceftazidime antibiotic) against three clinical isolates of *B. pseudomallei* and *E. coli* O157:H7. The experiments were performed by resazurin colorimetric assay and plate count. Data represent three independent experiments in triplicate.

Bacteria	Andro-AgNPs (µg/mL)		Ceftazidime (µg/mL)	
	MIC	MBC	MIC	MBC
*B. pseudomallei* 1206b*	0.5	8	32	128
*B. pseudomallei* H777**	0.5	8	256	1024
*B. pseudomallei* 316c***	0.5	8	256	1024
*E. coli* O157:H7	2	8	2	4

*CAZ non-resistant isolates.

**CAZ moderately resistant isolates.

***CAZ highly resistant isolates.

The results suggest that the andro-AgNPs have greater antimicrobial efficacy against *B. pseudomallei* than ceftazidime, particularly for the ceftazidime-resistant strains. As shown in Table 1, the MIC and MBC values of AgNPs toward *B. pseudomallei* were lower than ceftazidime by 64–512 fold and 16–128 fold, respectively. Moreover, *B. pseudomallei* showed a more sensitive response to andro-AgNPs (lower MIC values) when compared with *E. coli*. These might be due to the faster growth of *E. coli* via a binary fission process^35,36^. At the MIC concentration, bacterial growth is inhibited but some populations remain alive; subsequent rapid recovery of *E. coli* might then result in a higher measured MIC value.

The MIC and MBC values of the andro-AgNPs against the 3 isolates of *B. pseudomallei* herein are lower than previous reports of green-synthesized AgNPs. For example, the MIC and MBC values of starch and tannic acid-stabilized AgNPs against 3 isolates of *B. pseudomallei* were in range of 8–48 µg/mL and 16–128 µg/mL, respectively^36–38^. Since the AgNPs in those studies were similar in shape and size to the andro-AgNPs used here (spherical with average diameter in the range 10–20 nm), the enhanced antimicrobial efficiency of the andro-AgNPs against *B. pseudomallei* herein might be due to the andrographolide stabilization layer.

In any case the andro-AgNPs investigated herein display excellent antimicrobial activity against *B. pseudomallei*, including problematic ceftazidime-resistant strains, with a high concentration of bacteria (approximately 10^7^ CFU/mL) being susceptible to very low concentrations of AgNPs (0.5 µg/mL).

### Time-kill kinetics assay

**Figure 3:**
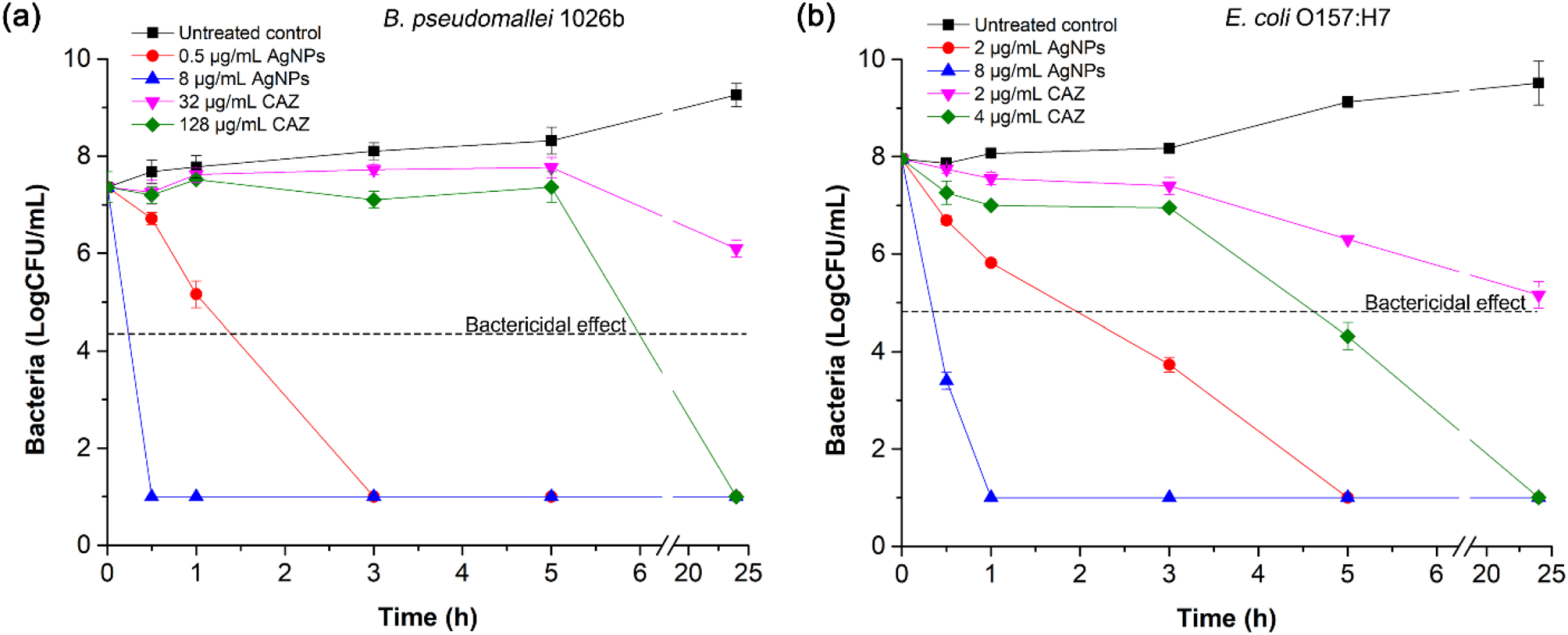
Killing time of antimicrobial agents (andro-AgNPs and ceftazidime (CAZ) antibiotic) against *B. pseudomallei* 1026b (**a**) and *E. coli* O157:H7 (**b**). The bacteria were incubated with antimicrobial agents at the concentration of MIC and MBC for 24 h (for andro-AgNPs, MIC and MBC are 0.5 and 8 µg/mL for *B. pseudomallei* 1026b, and 2 and 8 µg/mL for *E. coli* O157:H7. For CAZ, MIC and MBC are 32 and 128 µg/mL for *B. pseudomallei* 1026b, and 2 and 4 µg/mL for *E. coli* O157:H7). At time points of 0, 0.5, 1, 3, 5, and 24 h, 50 µL of each sample was taken for serial dilution and plate count. The bactericidal effect was defined as ≥ 3 log10 reduction compared to an untreated control. Data represent mean value ± SD (error bar) from two independent experiments in triplicate (n = 6).

To determine the optimum treatment time for andro-AgNPs against *B. pseudomallei*, kinetic time-killing assays were performed by plate count method for *B. pseudomallei* 1026b and *E. coli* O157:H7 (the latter again serving as a Gram-negative control). The bactericidal effect threshold was defined as ≥ 3 log10 reduction compared to the starting log CFU/mL. The results shown in Fig. 3 reveal that andro-AgNPs at the MIC and MBC values displayed bactericidal activity against *B. pseudomallei* 1026b and *E. coli* O157:H7 within 3 h, and within 30 min, respectively. These results are similar to previous studies that found AgNPs exhibited bactericidal activity within 30 min^37,38^. On the other hand, ceftazidime at the MIC concentration had no bactericidal effect. At the MBC concentration, ceftazidime displayed a bactericidal effect after 5 h and 3 h against *B. pseudomallei* 1026b and *E. coli* O157:H7, respectively. This is a typical killing time for an antibiotic such as ceftazidime.

The fact that *B. pseudomallei* 1026b had its growth inhibited and was completely killed faster than *E. coli* O157:H7 in the presence of andro-AgNPs might again be attributed to the faster growth of *E. coli* via binary fission^35,36^. Nevertheless both bacteria are highly susceptible to andro-AgNPs, with fast killing action within 30 min despite the high initial number of bacterial cells. This fast action may be useful to reduce the risk of bacterial resistance induction, however, more elaborate experimental evidence will be required to confirm this.

### Membrane neutralization

**Figure 4:**
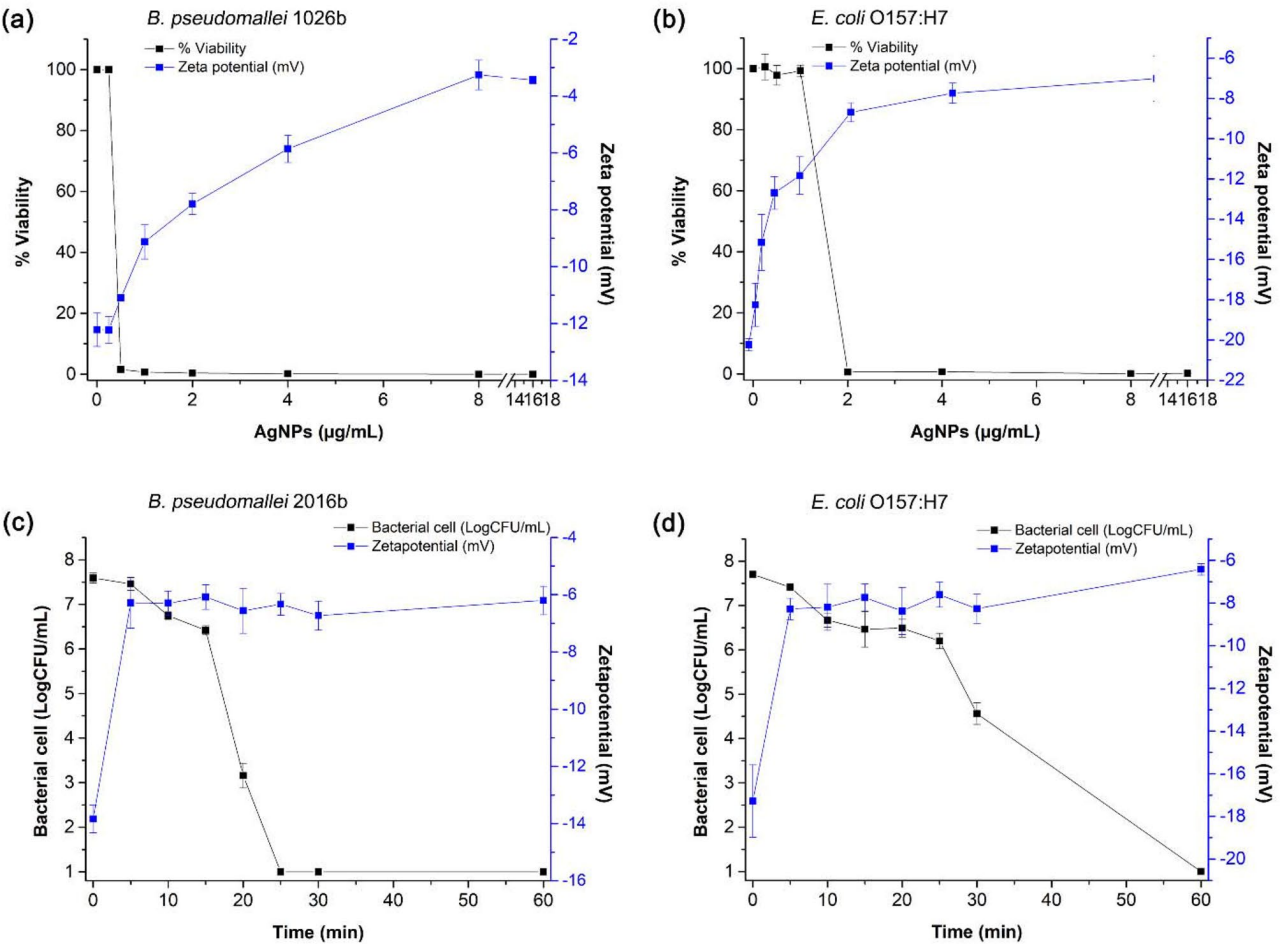
Effect of andro-AgNPs on bacterial viability and cell surface charge of *B. pseudomallei* 1026b (**a**) and *E. coli* O157:H7 (**b**). The bacteria were treated with andro-AgNPs concentrations in the range 0.25–16 μg/mL. The black line corresponds to the percentage of viable bacterial cells in the presence of increasing andro-AgNPs concentration, whereas the cell surface zeta potential is indicated by the blue line. Data represent the means ± standard errors for 6 samples. The correlation between cell membrane neutralization and cell viability over 60 min for *B. pseudomallei* 1026b (**c**) and *E. coli* O157:H7 (**d**) after introduction of 8 µg/mL of andro-AgNPs (1xMBC) at 37 °C. After incubation, the bacteria colony count and zeta potential were measured every 5 min. Data are represented as mean value ± standard error from two independent experiments carried out in triplicate (*n* = 6).

Surface charge neutralization on bacterial surfaces is an important mode of action for antimicrobial agents. Surface charge neutralization leads to increased surface permeability and eventually decreased cell viability^39^. Normally, adhesion and accumulation of AgNPs on the surface of the bacterial cell wall and membrane is a first step in the anti-bacterial mode of action of AgNPs^40^. This interaction may vary in the case of *B. pseudomallei* and *E. coli* as their outer membranes have different lipopolysaccharide (LPS) chemistry and thus different electrostatics. Specifically, Lipid A of *B. pseudomallei* is modified with 4-amino-4-deoxy-arabinose (Ara4N)^41^, leading to decreased net negative charge and a zeta potential closer to neutral compared to *E. coli*. To investigate bacterial surface charge and possible neutralization by the accumulation of andro-AgNPs at the membrane, zeta potential measurements were carried out. The effect of andro-AgNPs on bacterial surface charge, and its correlation with bacterial viability, is shown for *B. pseudomallei* 1026b and *E. coli* O157:H7 in Fig. 4a, b. The average zeta potential of the untreated *B. pseudomallei* 1026b and *E. coli* O157:H7 were − 12.22 ± 0.58 mV and − 20.40 ± 0.30 mV, respectively. This correlates with the known differences in membrane composition between *B. pseudomallei* and *E. coli* mentioned above, *B. pseudomallei* having a modified biphosphorylated disaccharide backbone of lipid A that is a highly conserved component of its lipopolysaccharide, the Ara4N residue being attached to the phosphate group^41,42^. This results in the overall cell surface charge of *B. pseudomallei* being less negative than in *E. coli*. The surface charge of *E. coli* found here is similar to previous findings^43^.

Importantly, as andro-AgNPs were introduced to the system, the bacterial surface zeta potential moved towards neutrality and was accompanied by decreasing cell viability for both strains. At the MIC, where cell viability (%) is almost zero, the surface charge of *B. pseudomallei* shifted from −12.22 to −11.10 ± 0.10 mV, and for *E. coli* from −20.4 to −11.84 ± 0.93 mV, respectively. Meanwhile, at the MBC, where all bacteria were killed, the surface charge of *B. pseudomallei* and *E. coli* shifted further to −3.26 ± 0.52 mV and −7.74 ± 0.50 mV, respectively. The drop in bacterial viability of *B. pseudomallei* corresponds well with the increase of the cell surface zeta potential upon addition of AgNPs, in agreement with previous reports^38^. Membrane neutralization was greater for *E. coli* than *B. pseudomallei*, attributed to the greater negative charge at the surface of *E. coli*^42^.

Next we determined the surface charge of the two strains, and their viability, as a function of time in the presence of andro-AgNPs. The results in Fig. 4c, d show that surface charge was nearly neutralized (reached *ca*. −6 mV) within 5 min for both *B. pseudomallei* and *E. coli* and then remained stable for 60 min. Over the same timeframe, there was a more gradual decrease in cell viability for both strains. This implies that andro-AgNPs show fast action, binding to bacterial cell surface in minutes, leading to decreased cell viability over a longer timeframe. The mechanism for this decreased cell viability over time might be a surface binding-induced increase in the probability of the andro-AgNPs entering the cells^39^. This certainly occurs faster for *B. pseudomallei* than *E. coli* (complete cell death within 25 and 60 min respectively).

### Silver ion (Ag^+^) release

Another proposed mode of antibacterial action due to AgNPs is their release of Ag^+^. AgNPs can continually release Ag^+^, which binds to the bacterial cell wall and cytoplasmic membrane and leads to disruption of the bacterial envelope^44^. After Ag^+^ uptake into cell, the interaction of Ag^+^ with sulfur and phosphorus can damage protein and DNA^40^. Furthermore, Ag^+^ can also induce reactive oxygen species (ROS) production that leads to cell membrane disruption and DNA modification^45^. We studied Ag^+^ release from the andro-AgNPs after incubation with bacteria using Inductively Coupled Plasma Optical Emission Spectrometry (ICP-OES).

**Figure 5:**
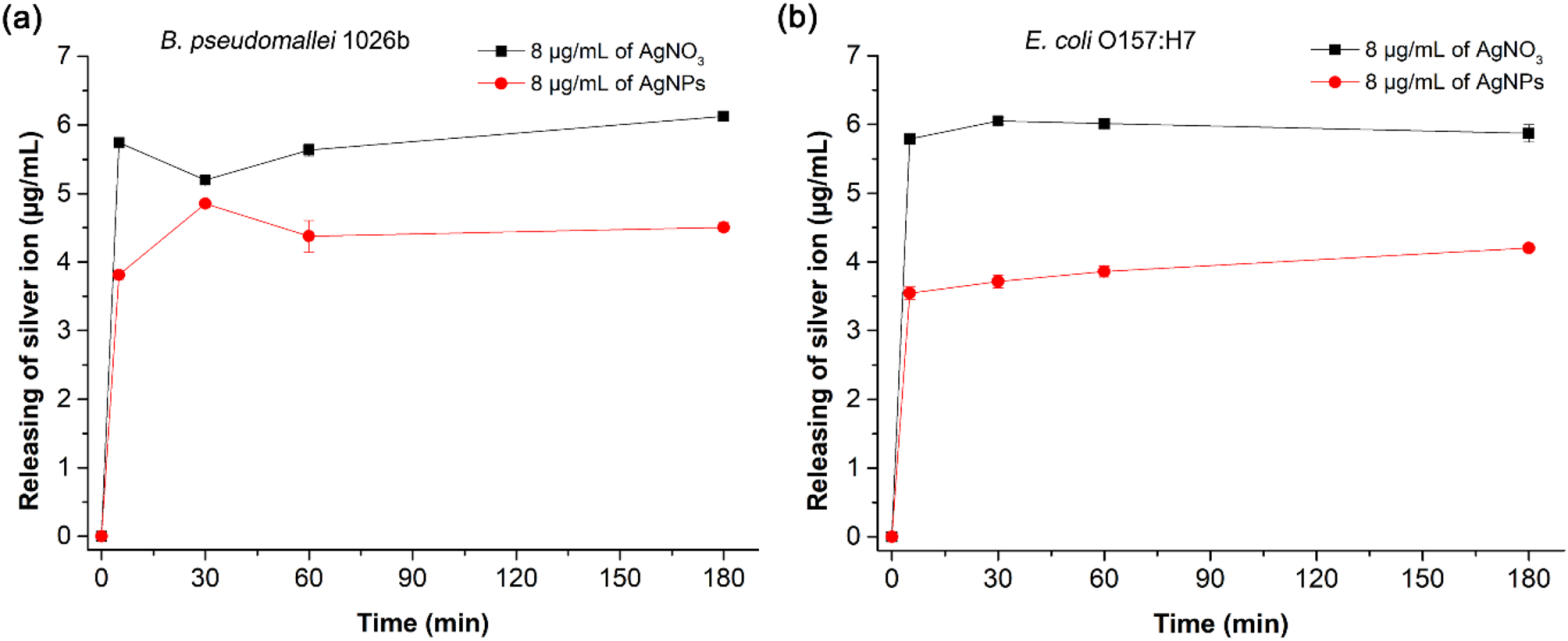
Silver ion release upon incubation of andro-AgNPs and AgNO_3_ with *B. pseudomallei* 1026b (**a**) and *E. coli* O157:H7 (**b**). The bacteria were treated with AgNPs or AgNO_3_ at 1xMBC for 0, 5, 30, 60, 180 min. The incubate was filtered using centrifugal membrane 30 K and the flow-through fraction examined for silver ion concentration by ICP-OES. Data represents the mean ± standard error from two independent experiments performed in triplicate (n = 6).

In Fig. 5, Ag^+^ release is measured over time after incubating the MBC of andro-AgNPs with *B. pseudomallei* 1026b and *E. coli* O157:H7. Ag^+^ release after incubation with the same concentration of AgNO_3_ is included as a reference. Ag^+^ release increased to 3.81 ± 0.06 µg/mL after 5 min incubation of andro-AgNPs with *B. pseudomallei* 1026b (Fig. 5a). Ag^+^ then continued to increase to 4.85 ± 0.06, 4.38 ± 0.22, and 4.51 ± 0.07 µg/mL at 30, 60, and 180 min incubation time respectively.

Similar trends are observed for Ag^+^ release by andro-AgNP-treated *E. coli* (Fig. 5b). Ag^+^ release increased to 3.54 ± 0.09 µg/mL after 5 min incubation, then continuously increased with incubation time, reaching 3.72 ± 0.09, 3.86 ± 0.08, and 4.20 ± 0.05 µg/mL at 30, 60, and 180 min, respectively. The Ag^+^ release pattern for AgNPs incubation was similar to that for AgNO_3_ in both bacterial strains. This suggests that Ag^+^ was present in a very large fraction compared to the starting concentration of AgNPs at 8 μg/mL.

Our results suggest that Ag^+^ release is a potential antimicrobial mode of action for AgNPs^46^. However other studies have concluded that Ag^+^ release was insignificant when incubating *B. pseudomallei* with AgNPs and that AgNPs mainly contributing to the killing action ^38^. Efficiency of Ag^+^ release from AgNPs can be affected by the reducing agent, surface stabilizer, and the synthesis method used. For example, Ag^+^ release from citrate capped-AgNPs was higher than for 11-mercaptoundecanoic acid-capped AgNPs^47^. The synthesis and stabilization of AgNPs with andrographolide in this work might promote greater Ag^+^ release than observed in previous studies. As andrographolide is a relatively small stabilizer, it might leave the metallic Ag surface open to oxidation under ambient conditions^47,48^. These results indicate that Ag^+^ could play an important role in the antimicrobial activity of andro-AgNPs, perhaps contributing to surface charge neutralization and decreasing of bacterial cell viability observed in the previous section (Fig. 4).

### Intracellular ROS production

Induction of reactive oxygen species (ROS) inside bacterial cells is another important mechanism by which AgNPs act on bacterial cells^46^. ROS induction by AgNPs and Ag^+^ lead to damage of biomolecules, including lipids, proteins, and DNA, leading to cell death^49^. Since the andro-AgNPs here strongly interact with *B. pseudomallei* 1026b and *E. coli* O157:H cell surfaces, and release significant amounts of Ag^+^, generation of ROS could be a significant factor in the anti-microbial action observed.

**Figure 6:**
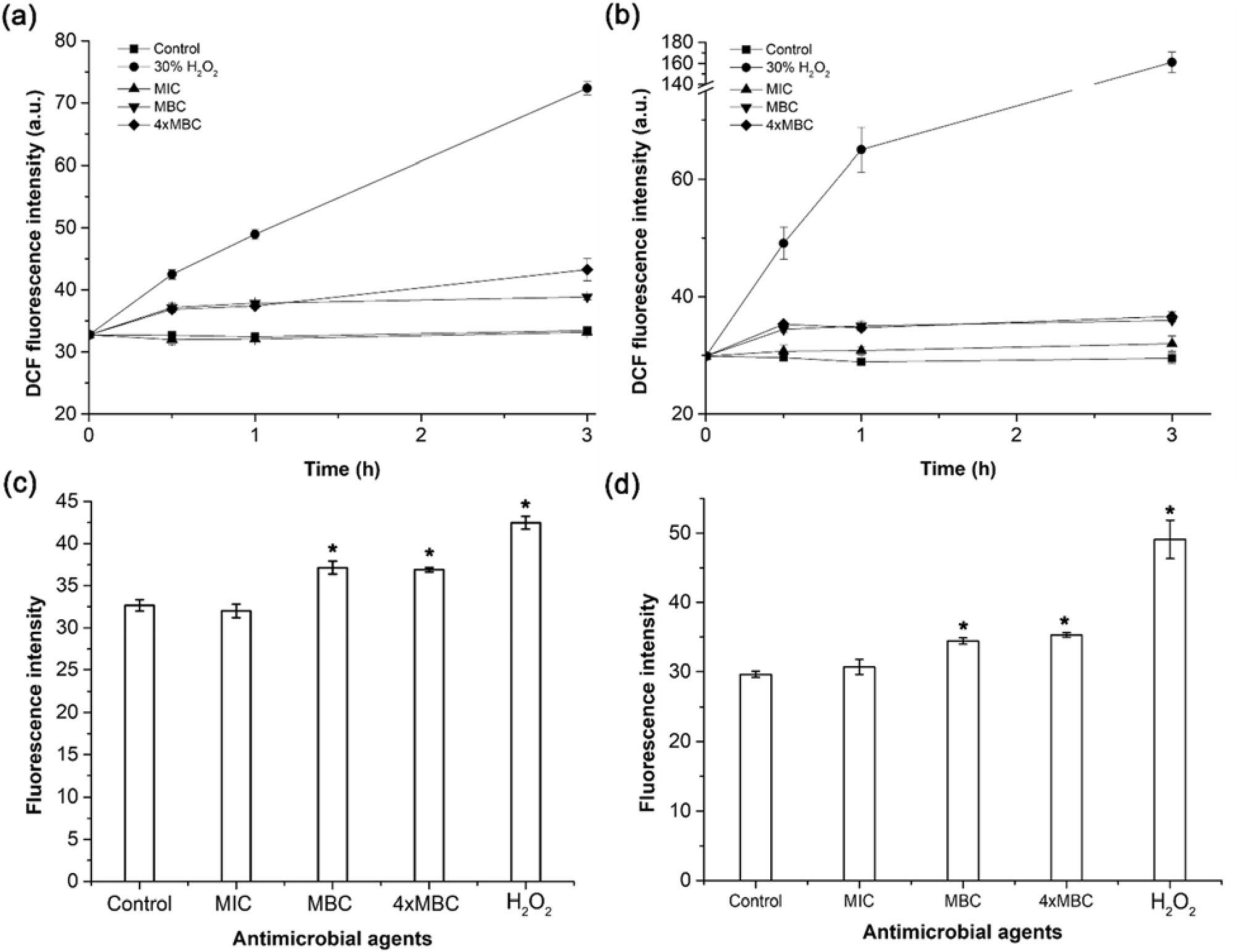
Kinetics of ROS induction in *B. pseudomallei* 1026b and *E. coli* O157:H7 after exposure to andro-AgNPs. All bacterial cells were treated with various concentrations of andro-AgNPs: 0 μg/mL (untreated control), MIC, MBC, and 4xMBC of AgNPs. 30% H_2_O_2_ was used as a positive control. Kinetics of induction of ROS in *B. pseudomallei* 1026b (**a**) and *E. coli* O157:H7 (**b**) and accumulated ROS at 30 min (**c**, **d**) were followed by DCF fluorescence. An asterisk (*) indicates significant difference in ROS level in comparison with untreated control (*p* < 0.01). Data represents the mean ± standard error from two independent experiments performed in triplicate (n = 6).

Herein, andro-AgNP-induced intracellular ROS generation was assessed for *B. pseudomallei* 1026b and *E. coli* O157:H7 using the pro-fluorescent oxidation-sensitive dye 2',7'-dichlorofluorescein diacetate (DCF-DA) as a ROS-sensing substrate (DCF-DA is oxidized by ROS to become the highly fluorescent 2′,7′-dichlorodihydrofluorescein, DCF). The kinetics of ROS induction at the MIC concentration of andro-AgNPs are shown in Fig. 6 and show no significant difference to an untreated control in *B. pseudomallei*, whereas for *E. coli* there is a significant increase in ROS at 1 h and 3 h of incubation (*p* < 0.05) (Fig. 6a, b). Resistance to ROS has been observed previously for *B. pseudomallei* and is thought to be a reason it can evade immune cells and survive in hosts^50^. At the 1xMBC and 4xMBC concentration of andro-AgNPs, the amount of ROS continuously increased with time, but took longer for *B. pseudomallei* than for *E. coli* (Fig. 6a, b). The DCF fluorescence intensity from the bacterial sample treated with andro-AgNPs at 1xMBC and 4xMBC concentration are clearly significantly higher than untreated control (*p* < 0.01) within 30 min of incubation (Fig. 6c, d). These results are similar to those of Pawinee et al.^41^ that found AgNPs can induce ROS generation inside *B. pseudomallei*. However, they reported increasing ROS after 3–4 h, whereas our andro-AgNPs show significant ROS generation within 30 min compared to untreated controls. ROS induction kinetics here correlate well with the membrane neutralization and Ag^+^ release kinetics (Figs. 4 and 5) that found increasing zeta potential and Ag^+^ release in large fraction within 30 min at the MBC. Therefore, both andro-AgNPs directly, and Ag^+^ release from them, might generate the intracellular ROS within the bacterial cell.

### Membrane integrity and morphological change of bacteria

**Figure 7:**
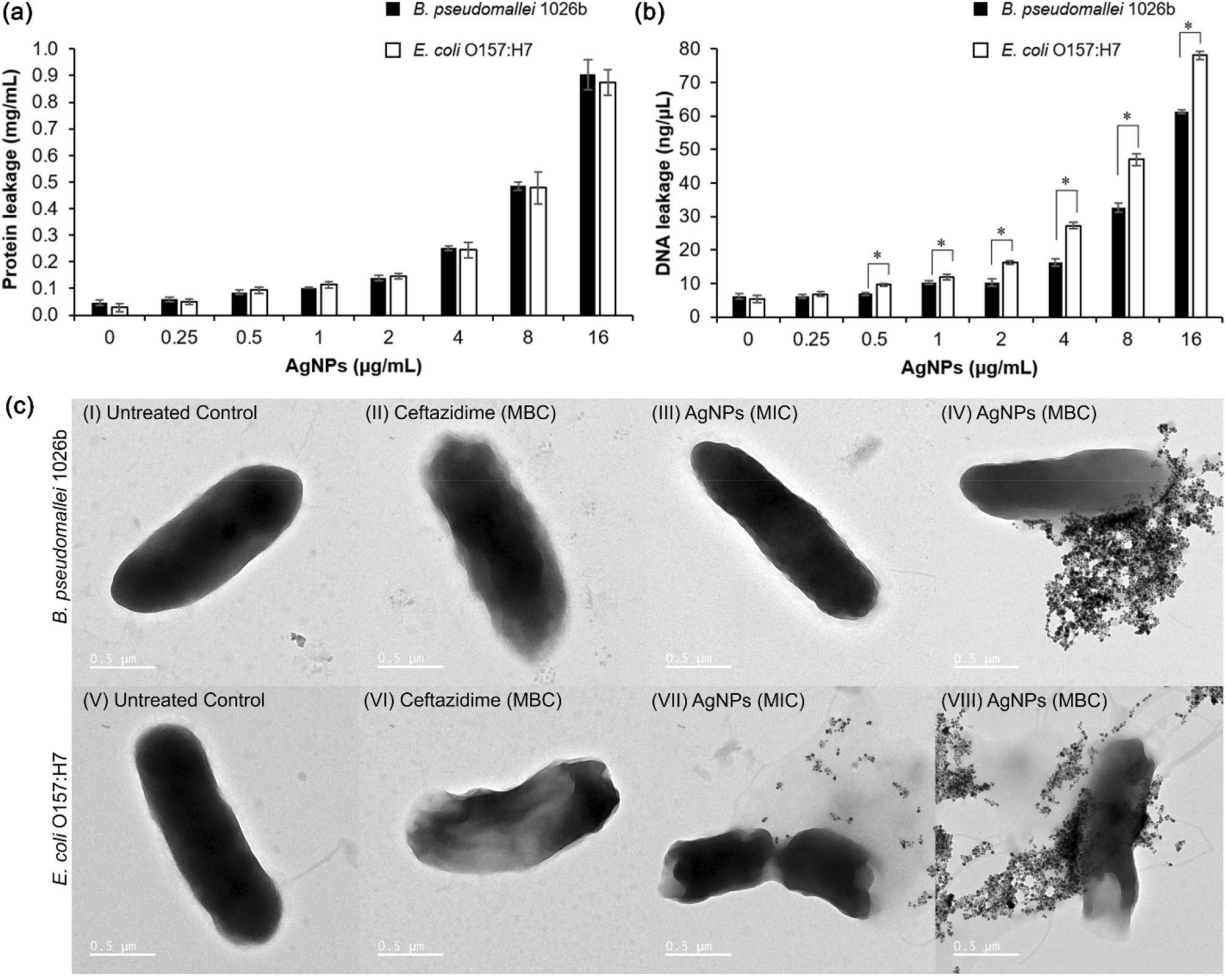
Membrane integrity and morphological change of bacteria in the presence of andro-AgNPs. Membrane integrity of *B. pseudomallei* 1026b and *E. coli* O157:H7 were evaluated by protein (**a**) and DNA leakage (**b**). The bacteria were incubated with various concentration of AgNPs for 1 h. Supernatant of protein and DNA leakage were kept after centrifugation and quantified by absorption spectroscopy at 280 nm and 260 nm, respectively. An asterisk (*) indicates significant difference in DNA leakage between *B. pseudomallei* 1026b and *E. coli* O157:H7 (*p* < 0.05) samples. Data represents the mean ± standard error from two independent experiments performed in triplicate (n = 6). (**c**) TEM image of cell morphological change of *B. pseudomallei* 1026b (I-IV) and *E. coli* O157:H7 (V-VIII). The bacteria were treated with ceftazidime at the concentration of MBC or AgNPs at MIC and MBC for 1 h and then observed by TEM. The same cells in Mueller Hinton broth served as a negative control.

Next, bacterial membrane integrity in the presence of andro-AgNPs was evaluated. Bacterial membrane damage can be caused by AgNPs, Ag^+^ and ROS^46^. Here we assessed membrane integrity by protein and DNA leakage from *B. pseudomallei* 1026b and *E. coli* O157:H7. As shown in Fig. 7a and b, protein and DNA leakage of both bacteria were increased when increasing concentrations of andro-AgNPs were added. There was no significant difference in leakage of protein concentration between *B. pseudomallei* 1026b and *E. coli* O157:H7. On the other hand, DNA leakage was greater in *E. coli* O157:H7 compared to *B. pseudomallei* 1026b. The apparently more robust membrane integrity of *B. pseudomallei* may be due to its cell envelope structure being more complicated than *E. coli*^51,52^. Interaction of AgNPs and Ag^+^ directly at the membrane, but also ROS induction, could be the mechanism of AgNPs-induced membrane integrity loss observed here^44,46,53^.

Morphological studies of the bacteria were undertaken using TEM imaging to further confirm bacterial membrane integrity loss upon exposure to andro-AgNPs. *B. pseudomallei* 1026b and *E. coli* O157:H7 were imaged after 1 h incubation with andro-AgNPs at the MIC and MBC concentration, and with ceftazidime as a positive control. As shown in Fig. 7c, the *B. pseudomallei* and *E. coli* cells in PBS buffer display smooth and intact surfaces. After treatment with andro-AgNPs and ceftazidime, morphological changes to the cell envelope were observed including membrane corrugations, cell disruption, and release of cytoplasmic components. At lower concentrations of andro-AgNPs, such as the MIC concentration, the NPs can penetrate the slime layer and interact with the bacterial cell surface (Fig. S4). This caused roughening of the *B. pseudomallei* cell surface (Fig. 7c (III)), while cell burst occurred for *E. coli* in the same conditions (Fig. 7c (VII). When the bacterial cells were treated at the MBC concentration of AgNPs, extensive adhesion and accumulation of AgNPs at the bacterial cell surface was observed (Fig. 7c (IV, VIII) and Fig. S5). This leads to denaturing of the cell membrane and eventually cell death^46^. Our results agree with previous work showing that AgNPs accumulate on the outer surface of *B. pseudomallei*^38^. We find that andro-AgNPs-treated *E. coli* cells exhibited greater cell burst frequency compared to *B. pseudomallei* cells which showed only membrane leakage and release of cytoplasmic components. Again, this difference is attributed to the more complicated structure and higher rigidity of the *B. pseudomallei* cell envelope^42,54^ and correlates with the protein and DNA leakage experiments (Fig. 7a and b). The TEM imaging studies confirm that andro-AgNPs interact with bacterial cell surfaces and cause membrane damage and leakage of cytoplasmic components.

### Model of the proposed mode of action of andro-AgNPs against B. pseudomallei

**Figure 8:**
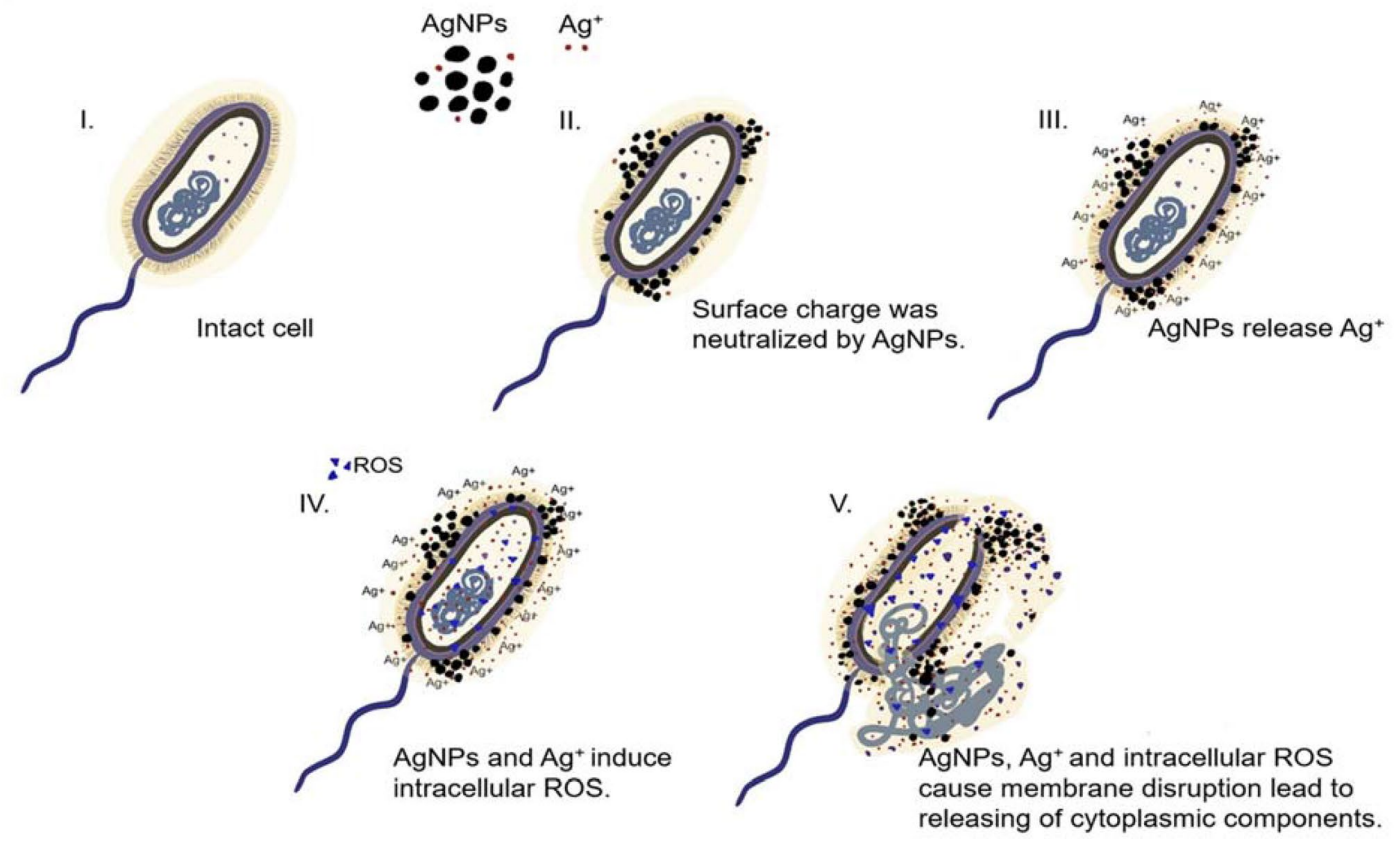
The proposed mode of action of andro-AgNPs against *B. pseudomallei*. (**I.**) Untreated bacterial cell with a smooth and intact surface. (**II.**) Once andro-AgNPs are added, bacterial cell surface charge is neutralized due to AgNP binding. (**III.**) After binding of AgNPs to cell surface, Ag^+^ is released in large fraction locally. (**IV.**) AgNPs and Ag^+^ induce intracellular ROS that promotes biomolecule (protein, DNA, lipid etc.) damage and a disrupted bacterial membrane. (**V.**) Eventually, bacterial cell death occurs by the combined action of AgNPs, Ag^+^ and generation of intracellular ROS. This figure was drawn by Saengrawee Thammawithan.

The proposed mode of action of andro-AgNPs against *B. pseudomallei* evidenced from the studies herein is shown schematically in Fig. 8. Once andro-AgNPs are incubated with bacterial cells, surface binding occurs, neutralizing the membrane charge within 5 min (Fig. 4). This implies effective adhesion and accumulation of andro-AgNPs on the cell surface (confirmed by Fig. 7c), with consequent structural changes. The andro-AgNPs also release Ag^+^ in large fractions after 5 min of incubation and continue this release for 3 h (Fig. 5). Ag^+^ likely also adhere to the cell wall and cytoplasmic membrane, enhancing the permeability of the cytoplasmic membrane and the destruction of the bacterial envelope. Both andro-AgNPs and Ag^+^ could induce the observed generation of intracellular ROS that promoted biomolecule (protein, DNA, lipid etc.) damage and further disruption of the bacterial membrane (Fig. 6). Eventually, the bacteria exhibit membrane leakage and releasing of cytoplasmic components leading to death (Fig. 7).

## Conclusion

In the present study, andrographolide stabilized-AgNPs were evaluated for their biological activity including cytotoxicity to mammalian cells, antimicrobial activity, and mode of action against *B. pseudomallei*. The andro-AgNPs display excellent antibacterial effect against *B. pseudomallei,* including ceftazidime antibiotic-resistant isolates, with MIC and MBC values 1–3 orders of magnitude lower than ceftazidime, and 1–2 orders of magnitude lower than for starch- and tannic acid-stabilized AgNPs. Moreover, the andro-AgNPs showed low cytotoxicity towards both normal and cancerous mammalian cells. The mode of anti-microbial action of andro-AgNPs involves binding and charge neutralization at the membrane surface, and subsequent Ag^+^ and ROS generation, that may all contribute together to the killing of the cell. These effects occur faster than for AgNPs treatments in previous studies, suggesting a role for the andrographolide in improving both antimicrobial effect and efficiency when employed as a AgNP stabilizer. In summary, plant-extracted andrographolide stabilized-AgNPs are a promising alternative antimicrobial agent for combating ceftazidime-resistant *B. pseudomallei* and may have a role in future melioidosis treatments.

## Materials and methods

### Materials

Silver nitrate (AgNO_3_, 99.9%) was purchased from POCH™, Poland. Andrographolide was purchased from Khaolaor laboratories Co., Ltd., Samut Prakan, Thailand. Ceftazidime antibiotic was purchased from Reyoung Pharmaceutical Co., Ltd, China. Mueller Hinton Broth was purchased from HiMedia, India. 3-(4,5-dimethylthiazolyl-2)-2,5 diphenyltetrazolium bromide (C_18_H_16_BrN_5_S, 98%) was purchased from AppliChem GmbH, Darmstadt, Germany. 7-hydroxy-3H-phenoxazine-3-one 10-oxide sodium salt (C_12_H_6_NNaO_4_, ~ 80%) and 2′,7′-Dichlorofluorescin diacetate (C_24_H_16_Cl_2_O_7_, ≥ 97%) were purchased from Sigma-Aldrich, St. Louis, Missouri, the United States. Glutaraldehyde (C_5_H_8_O_2_, 25% in H_2_O) were purchased from Polysciences, Inc., Warrington, Pennsylvania, the United States. Ethyl alcohol (C_2_H_6_O, 99.9%) was purchased from Quality Reagent Chemical (QReC), New Zealand.

### Preparation of andrographolide broth

Andrographolide (180 mg) was dissolved in 60 mL of deionized water and stirred at room temperature for 1 h. Then, the andrographolide solution was filtered by Whatman paper number 1. Filtrate was collected and filtered again by micro filter to obtain the andrographolide broth.

### Synthesis of andrographolide stabilized-silver nanoparticles

Andrographolide broth (3 mL) was dissolved in 24 mL of deionized water. After that, 3 mL of 20 mM silver nitrate was added and stirred vigorously at room temperature for 3 h. The color of the solution changed from yellow to reddish brown upon formation of the andrographolide stabilized-AgNPs.

### Characterization of andrographolide-stabilized silver nanoparticles

The characterization of andrographolide-stabilized silver nanoparticles were performed by UV − visible spectrophotometeric analysis, transmission electron microscope (TEM), Fourier transform infrared (FTIR) spectroscopy, and X-ray diffractometry (XRD)^55,56^. Absorption spectra of andro-AgNPs colloidal solutions were taken over the wavelength range 200–800 nm. For TEM studies, andro-AgNPs colloidal solutions were dropped onto carbon film-supported copper grids and observed by a FEI/TECNAI G2 20 TEM (FEI Company, Oregon, USA). FTIR spectra were recorded in the solid state by an Attenuated Total Reflectance (ATR) FTIR spectrophotometer using a standard Pike ATR cell (Model: Bruker TENSOR 27, Netherland) over the range 4000–600 cm^–1^. The dried powder of andro-AgNPs was further analyzed by X-ray diffraction (PANalytical Empyrean powder diffractometer) with an X-ray source of CuKα radiation (λ = 0.15418 Å). The analysis was carried out by a conventional 2theta scan over the range 20–80 degrees.

### Bacterial strain and growth conditions

Three clinical isolates of *B. pseudomallei* were kindly provided by the Melioidosis Research Center, Khon Kaen University (Khon Kaen, Thailand). *E. coli* O157:H7 was obtained from the Protein and Proteomics Research Group, Khon Kaen University. These bacteria were stored at –70 °C in 20% glycerol until used. *B. pseudomallei* were streaked on Ashdown’s medium (a selective culture medium for the isolation and characterization of *B. pseudomallei*) and then cultured at 37 °C for 48–72 h. *E. coli* O157:H7 were streaked on Mueller Hinton agar (MHA) and incubated at 37 °C for 24 h. The colonies of these bacteria were picked and inoculated in 5 mL of Mueller Hinton broth (MHB) at 37 °C overnight. Then, bacteria were sub-cultured in 5 mL of the same medium at 37 °C in a 180 rpm shaker-incubator for 3 h to yield a mid-logarithmic growth phase culture^36^.

### Cytotoxicity assays

The cytotoxicity of AgNPs was performed by the 3-(4,5-dimethylthiazolyl-2)-2,5 diphenyltetrazolium bromide (MTT) assay, which gives information on the cell proliferation rate, and the reduction in cell viability when metabolic events lead to apoptosis or necrosis. MTT, a yellow compound, is reduced by mitochondrial dehydrogenases to an insoluble purple formazan in living cells. A solvent, such as dimethyl sulfoxide, is added to dissolve the formazan crystal. The formazan concentration is then quantified by absorption spectroscopy in the range 500–600 nm. Vero cell and MCF-7 cell lines were employed as representative normal and cancerous mammalian cell lines, respectively. The cells were prepared in a 96-well plate. After 24 h, 100 μg/mL of andro-AgNPs was added to the plate. Cells without treating agents served as the control. To evaluate their viability, the cells were incubated and examined after 24, 48, and 72 h. After this period of treatment, 10 μL of 5 μg/mL MTT stock solution was added to each well and incubated for 4 h at 37℃. The medium was removed, and 150 μL of dimethyl sulfoxide (DMSO) was added into each well to dissolve the formazan crystals. The solution was measured at 570 nm by a Varioskan™ LUX multimode microplate reader (Thermo Scientific, USA)^57^. Experiments were performed in triplicate. Cell viability was calculated using the following formula:

$$\mathrm{Cell\; viability} \left(\%\right)=\frac{\mathrm{Absorbance\; of\; treated\; cell}}{\mathrm{Absorbance\; of\; control\; cell}}\times 100$$

### Antimicrobial susceptibility

Bacterial cells were prepared to OD_630_ = 0.02 (10^7^ CFU/mL) with MHB broth. 50 µL of AgNPs or CAZ in range of 0–1,024 µg/mL were prepared in a 96-well plate. Bacterial suspension was added in equal volume to the wells. Plates were then incubated at 37 °C for 18–24 h. After overnight incubation, 10 µL of 0.01% resazurin solution (7-hydroxy-3H-phenoxazine-3-one 10-oxide; Sigma-Aldrich) was added to each well and incubated at 37 °C for 2–3 h. A color change was observed. The lowest concentration before the color change was determined as the MIC, the blue color represented no growth of bacteria and the pink one indicates bacterial survival^37^. The MBC value was determined when there was no colony growth from the directly plated contents of the wells. The tests were performed in three independent experiments in triplicates.

### Time-kill kinetic assay

Overnight grown culture of *B. pseudomallei* 1026b and *E. coli* O157:H7 were diluted in fresh MH broth and allowed to grow to obtain OD 630 nm of 0.6. The bacteria were diluted in same media to OD 630 of 0.02. The dilution of bacteria were incubated at 37 °C with AgNPs or CAZ at MIC and MBC concentration. At interval time point of 0, 0.5, 1, 3, 5 and 24 h, 50 µL of solution was taken and diluted10-fold in PBS buffer. 10 µL of each dilution were dropped on MH agar and then incubated at 37 °C overnight. Bacterial colonies were counted and evaluated for bactericidal effect. The bactericidal effect is defined as greater than 3 log10-fold decrease in colony forming units^37^. Bacteria in MHB served as an untreated control. The tests were performed in two independent experiments in triplicates.

### Membrane neutralization

Following a previous method^38^, overnight growths of *B. pseudomallei* 1026b and *E. coli* O157:H7 were washed twice with sterile deionized water. The bacteria were diluted to 10^7^ CFU/mL (O.D.600 = 0.02). Then, the bacteria were incubated with andro-AgNPs at the concentration of 0, 0.25, 0.5, 1, 2, 4, 8, 16 µg/mL for 30 min. Zeta potential of bacterial cells were measured by using Zetasizer Nano ZS (Malvern Panalytical, Malvern, Worcestershire, England). The kinetics of membrane neutralization were obtained by incubating bacterial cells with andro-AgNPs at the MBC. At time intervals of 0, 5, 10, 15, 20, 25, 30, and 60 min of treatment, the bacterial suspensions were examined by the Zetasizer.

### Silver ion release

Silver ion (Ag^+^) release studies were performed using inductively coupled plasma optical emission spectrometry (ICP-OES)^38^. Briefly, mid-log phases of *B. pseudomallei* 1026b and *E. coli* O157:H7 were washed and re-suspended in deionized water. The bacteria were incubated with AgNPs or AgNO_3_ at the MBC at 37 °C. At 0, 5, 30, 60, and 180 min incubation, solution was taken, particles removed by centrifuge with an Omega membrane 30 K (Pall corporation, Port Washington, NY, U.S.A.) at 4,000 rpm for 15 min. The flow-through fraction was analysed by ICP-OES (PerkinElmer Optima 2100 DV, Markham, ON, Canada) for Ag^+^. The assay was completed two independent experiment in triplicate.

### Intracellular ROS production

Presence of reactive oxygen species (ROS) in *B. pseudomallei* and *E. coli* O157:H7 was estimated using 2′,7′-Dichlorofluorescin diacetate (DCFH-DA)^58^. This molecule is non-fluorescent but becomes the highly fluorescent 2’,7’-dichlorofluorescein (DCF) when oxidized by the action of intracellular ROS and other peroxides. In this study, the bacteria were washed two times in PBS buffer and re-suspended in same buffer. Then, 10^7^ CFU/mL of the bacteria were incubated with 10 µM of DCFH-DA at 37 °C for 1 h. After that, excess dye was washed away twice with sterile DI water. The bacteria were then incubated with andro-AgNPs for 3 h. At 0, 0.5, 1, and 180 min of treatment, the solution fluorescence was measured by SpectraMax M5 fluorescence microplate reader (Molecular devices, California, USA) at the excitation/emission wavelengths of 488/525 nm.

### Membrane integrity

Protein and DNA leakage were determined as a measure of integrity of the bacterial membrane. Experiments were performed according to a previous study with some modifications^59^. Briefly, overnight growths of *B. pseudomallei* and *E. coli* O157:H7 were washed two times with sterile PBS buffer. The bacteria were diluted and incubated with andro-AgNPs at 37 °C for 1 h. After incubation, the bacteria were centrifuged at 4,000 rpm for 15 min. The supernatant was kept. Protein and DNA concentration were measured by NanoDrop at 280 nm and 260 nm, respectively. The experiments were performed in two independent experiments in triplicates.

### Bacterial cell morphological change

Overnight growths of *B. pseudomallei* 1026b and *E. coli* O157:H7 were washed twice with sterile PBS buffer. The bacteria were prepared to 10^7^ CFU/ml (O.D.600 = 0.02) with the same buffer. One hundred microliters of diluted bacterial suspension was then incubated with 100 µl of andro-AgNPs or ceftazidime (to final concentrations at MIC and MBC) in a 96-well plate. The mixture was incubated at 37 °C for 1 h. Then, the 200 µl of cell suspension was centrifuged at 4,000 rpm for 15 min. The collected cell pellet was washed twice with sterile phosphate-buffered saline. Then, the bacterial cells were fixed with 2.5% glutaraldehyde overnight and dehydrated in a gradient of ethanol (30%, 50%, 70%, and 90%) for 10 min followed by rinsing in 100% ethanol twice. Ten microliters of cell suspension was then gently dropped on the TEM grid and dried overnight in a desiccator. The cell images were visualized and photographed by transmission electron microscope (FEI/TECNAI G2 20, FEI Company, Oregon, USA)^60^.

## Supplementary Information

Supplementary Information.
