# Supplementary material for: Andrographolide stabilized-silver nanoparticles overcome ceftazidime-resistant Burkholderia pseudomallei: study of antimicrobial activity and mode of action
Source: Sci Rep. 2022 Jun 23;12:10701. doi: 10.1038/s41598-022-14550-x (PMC9226156; doi:10.1038/s41598-022-14550-x)
Supplement: Supplementary file 1 — Supplementary Information. [file 41598_2022_14550_MOESM1_ESM.docx]

**Supplementary Information**

**Andrographolide stabilized-silver nanoparticles overcome ceftazidime-resistant *Burkholderia pseudomallei*: study of antimicrobial activity and mode of action**

Saengrawee Thammawithan^1,2,3^, Chanon Talodthaisong^3^, Oranee Srichaiyapol^1^, Rina Patramanon ^1,2^, James Andell Hutchison, Sirinan Kulchat ^3*^

^1^*Department of Biochemistry, Faculty of Science, Khon Kaen University, Khon Kaen 40002, Thailand*

^2^*Protein and Proteomics Research Center for Commercial and Industrial Purposes, Khon Kaen University, Khon Kaen 40002, Thailand*

*^3^Materials Chemistry Research Center, Department of Chemistry and Center of Excellence for Innovation in Chemistry, Faculty of Science, Khon Kaen University, Khon Kaen 40002, Thailand*

*^4^School of Chemistry, The University of Melbourne, Parkville, VIC, 3010, Australia*

* Corresponding author. Sirinan Kulchat

E-mail: sirikul@kku.ac.th

**FTIR spectroscopy**

The functional groups of pure andrographolide, commercially-sourced *A. paniculata*-derived andrographolide capsules, and of our as-synthesized Andro-AgNPs, were investigated using FTIR spectroscopy (Fig. S1). The spectrum of pure andrographolide shows peaks at 3300 cm^-1^ and 2900 cm^-1^ due to the presence of O-H and C-H stretches, respectively, while peaks at 1600 cm^-1^, 1400 cm^-1^ and 1050 cm^-1^ are attributed to stretches of C=O, C=C, and the C-O-C of the lactone ring respectively. The spectrum of the *A. paniculata*-derived andrographolide capsules shows strong peaks at similar energies, however the 1600 cm^-1^ attributed to C=O is relatively strongly suppressed. In the case of andro-AgNPs, again, similar characteristic peaks appear that were observed for isolated andrographolide. However, the intensity of the peaks at 1350 cm^-1^ (O-H bending) and 1050 cm^-1^ (C-O stretching) are suppressed, potentially due to interaction between andrographolide and the Ag^0^ surface in andro-AgNPs. Nevertheless, these results strongly suggest that andrographolide is bound to the surface of the AgNPs in our as-synthesized andro-AgNPs.

**
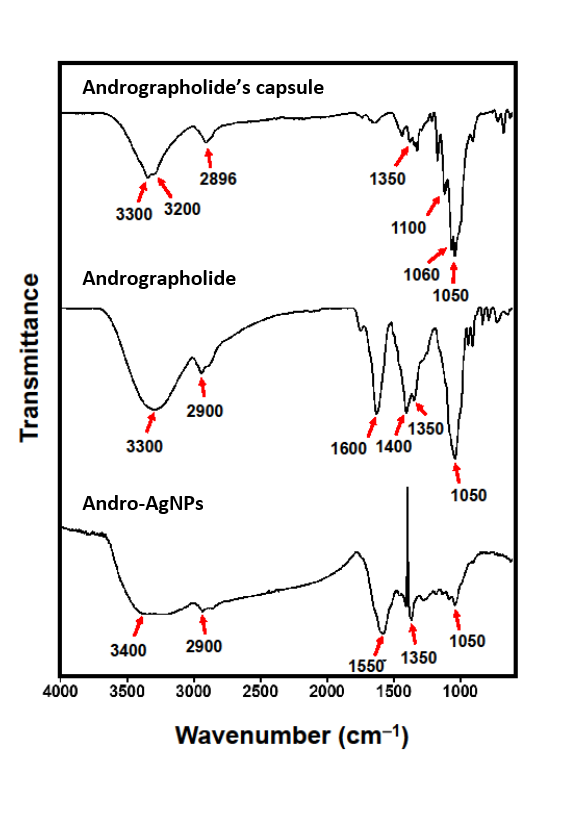
**

**Figure S1.** FTIR spectra of purified andrographolide (‘Andrographolide’), of *A. paniculata*-derived andrographolide capsules (‘Andrographolide capsule’), and of our as-synthesized andro-AgNPs (‘Andro-AgNPs’).

**X-ray diffraction (XRD) studies**

The XRD pattern of the andro-AgNPs is shown in Fig. S2. The pattern shows four sharp and well-defined diffraction lines at 2θ = 38.23°, 46.39°, 64.65° and 77.44°, which can be assigned to (111), (200), (220) and (311) planes respectively, indicating the silver nanoparticles are face-centered cubic (fcc) metallic silver. The well-defined and intense peaks confirm silver nanoparticle’s excellent crystallinity. The crystallite size was calculated using the Scherrer equation.

*D*_XRD_ = 0.94 *λ* /*β* Cos*θ*

where *D* is the average crystallite size perpendicular to the reflecting planes, λ is the X-ray wavelength (*λ* = 1.5418 Å), *β* is the line broadening at the full width at half maximum (FWHM) in radians, and *θ* is the Bragg angle in degrees. The peak of the (111) plane was used for the calculations, giving an average crystallite size of 3.82 nm from the FWHM of the peak.

**
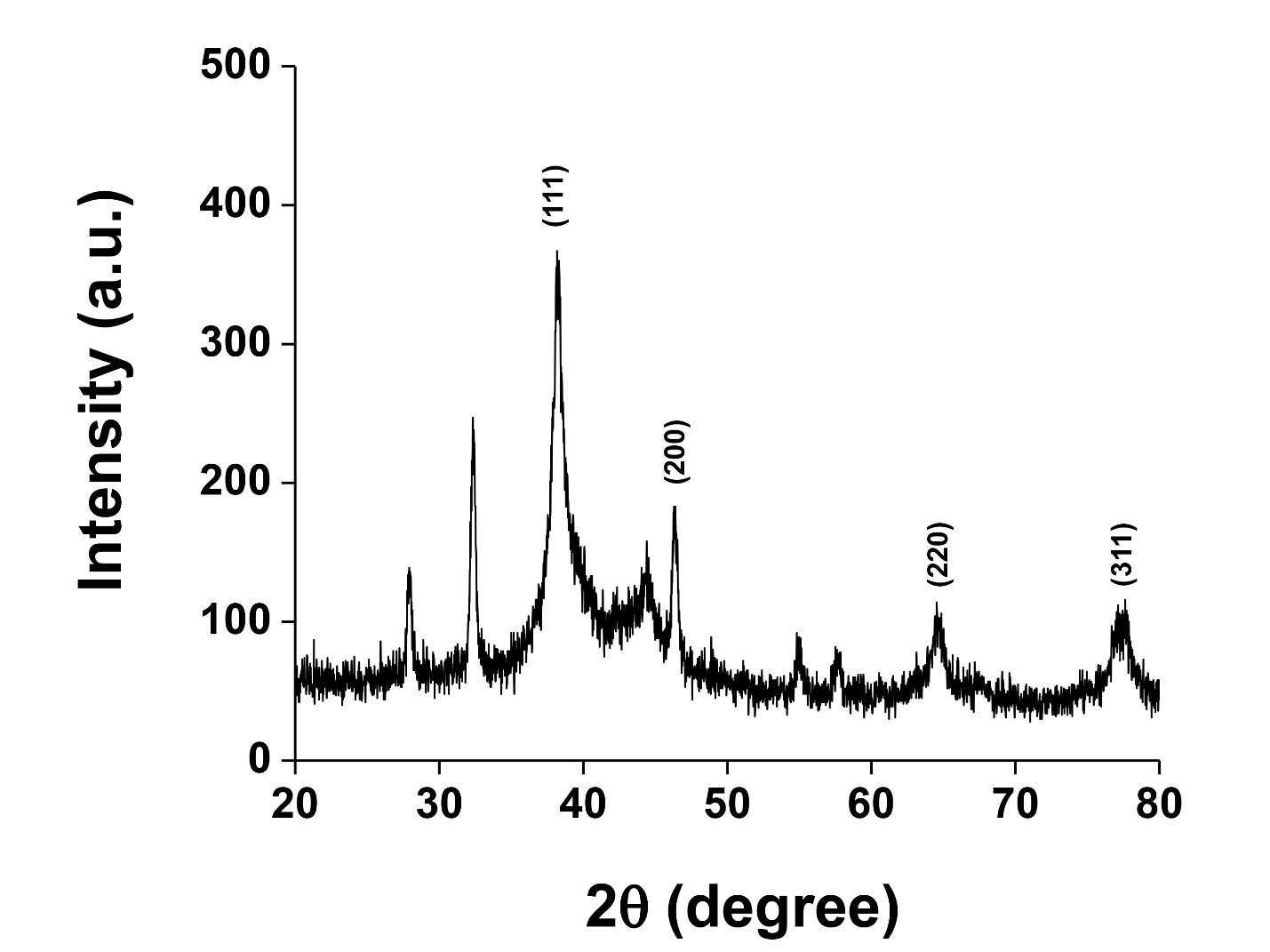
**

**Figure S2.** XRD pattern of the as-synthesized andro-AgNPs.


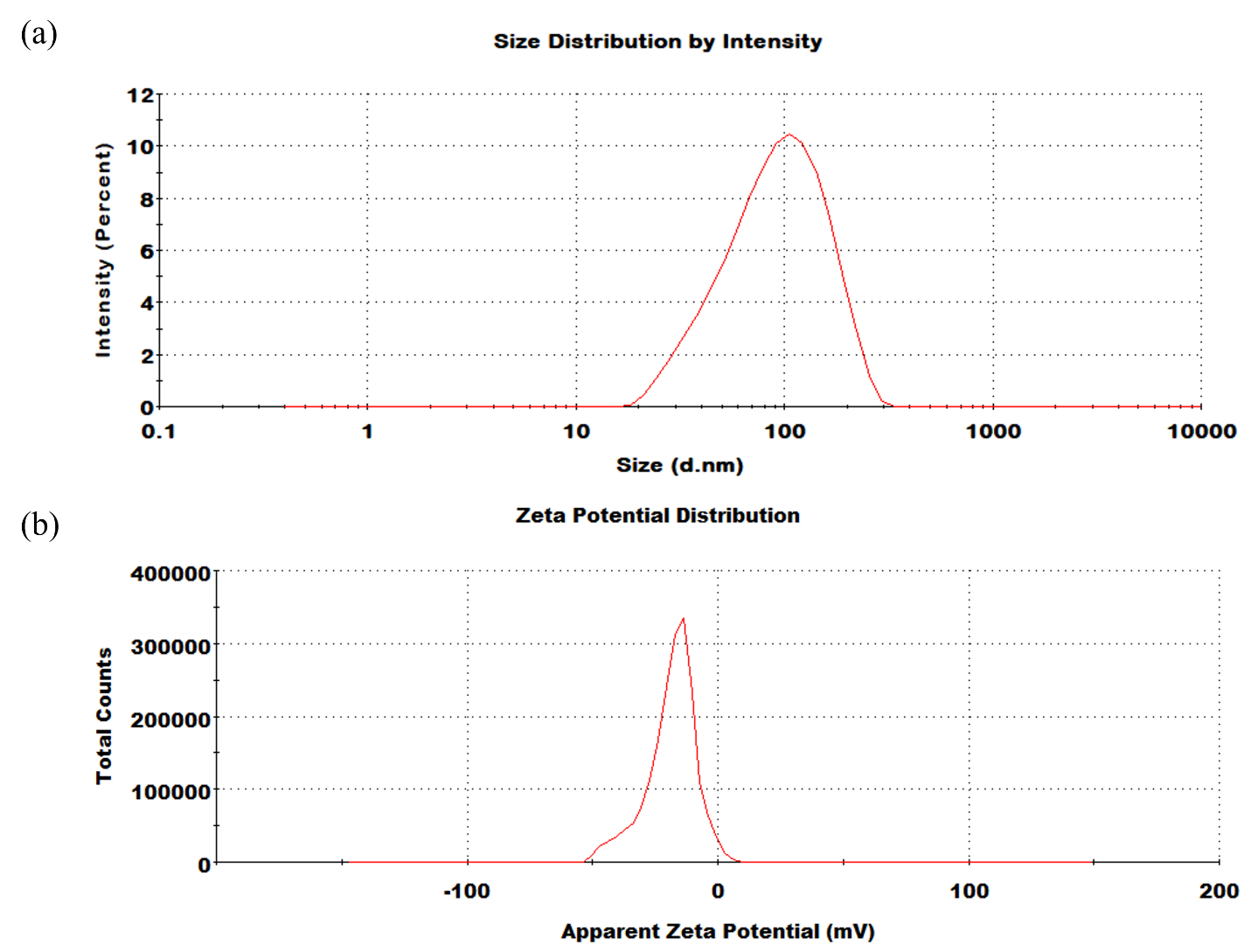


**Figure S3.** Physicochemical characterization of silver nanoparticles: (a) size distribution graph of AgNPs at 93.12 ± 3.76 nm with a poly-dispersity index (PDI) of 0.339 were obtained from Dynamic Light Scattering (DLS); and (b) zeta potential of AgNPs at -17.26 ± 0.99 mV.


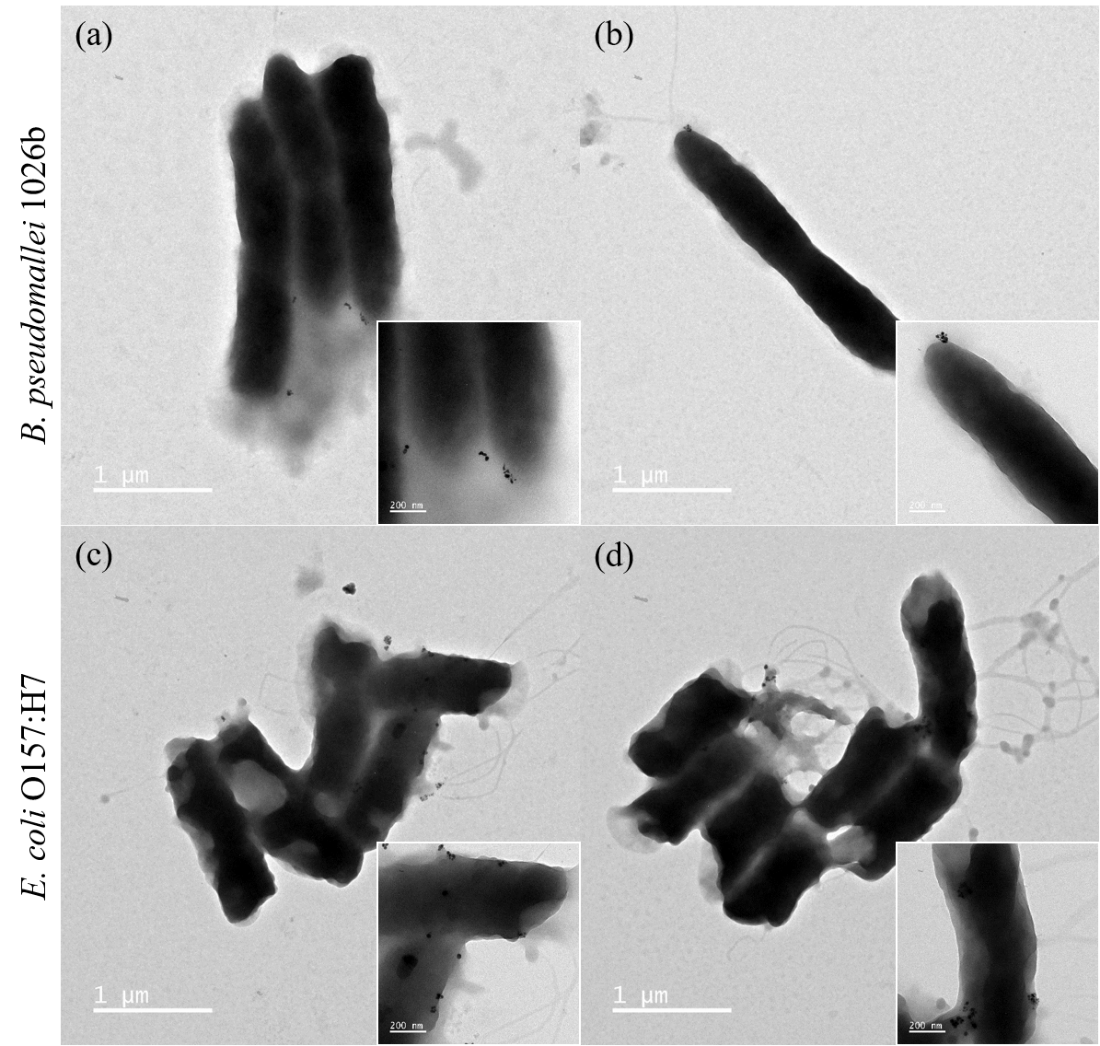


**Figure S4.** TEM images of andro-AgNPs on bacterial cells and cell morphological change of *B. pseudomallei* 1026b (a-b) and *E. coli* O157:H7 (c-d). The bacteria were treated with the AgNPs at the MIC concentration for 1 h. After incubation, bacteria were observed by using TEM. Insets, higher magnification showing the penetration of the slime layer and accumulation of andro-AgNPs on the cell membrane.


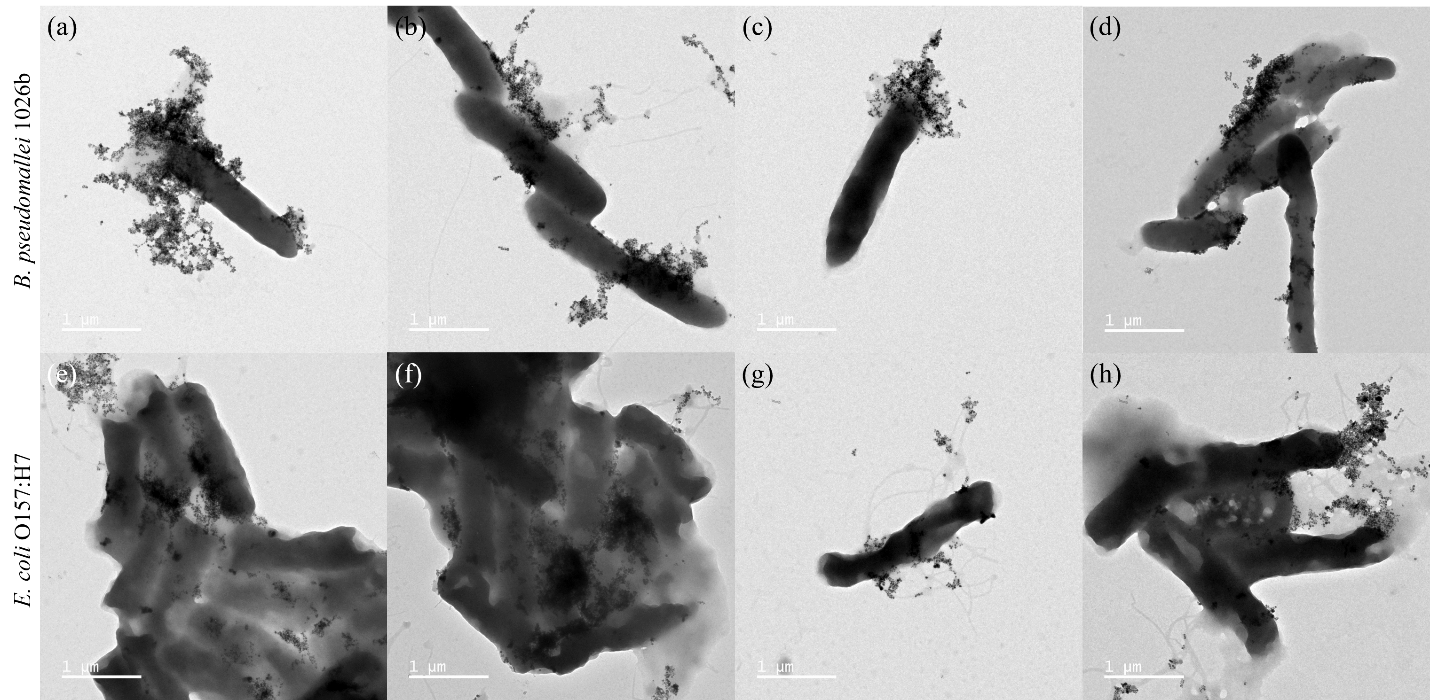


**Figure S5.** TEM image of cell morphological change of *B. pseudomallei* 1026b (a-d) and *E. coli* O157:H7 (e-h) upon exposure to andro-AgNPs at the MBC. Adhesion and accumulation of AgNPs on bacterial cells are observed. The bacteria were treated with andro-AgNPs at the MBC concentration for 1 h. After incubation, bacteria were observed by using TEM.
